# Effect of Kuijie Granule on the Expression of TGF-*β*/Smads Signaling Pathway in Patients with Ulcerative Colitis

**DOI:** 10.1155/2016/2601830

**Published:** 2016-02-25

**Authors:** Xinjie Xu, Chunhua Xu, Shakir M. Saud, Xiaoming Lu, Lei Liu, Li Fang, Xiaowei Zhang, Jiangong Hu, Weidong Li

**Affiliations:** ^1^Traditional Chinese Medicine Department, Affiliated Hospital of Taishan Medical University, Shandong 271000, China; ^2^Gastroenterology Department, Chengdu Second People's Hospital, Sichuan 610017, China; ^3^Department of Cell Biology and Molecular Medicine, Rutgers New Jersey Medical School, Newark, NJ 07103, USA; ^4^Gastroenterology Department, Liaocheng Second People's Hospital, Shandong 252600, China; ^5^Pathology Department, Affiliated Hospital of Taishan Medical University, Shandong 271000, China; ^6^Dermatology Department, Tai'an Maternal and Child Health-Care Hospital, Shandong 271000, China; ^7^Gastroenterology Department, Affiliated Hospital of Taishan Medical University, Shandong 271000, China; ^8^Department of Infectious Diseases, Guang'anmen Hospital, China Academy of Chinese Medical Sciences, Beijing 100053, China

## Abstract

The dysregulation of TGF-*β*/Smads signaling pathway has been postulated to contribute to the development of ulcerative colitis (UC) and the manifestation of clinical symptoms. Kuijie Granule is a prescription medicine used clinically in China to alleviate the symptoms associated with UC. To evaluate whether the clinical benefit of Kuijie Granule is associated with TGF-*β*/Smads signaling, we measured the expression levels of TGF-*β*/Smads signaling proteins (TGF-*β*1, TGF-*β*RII, Smad2, Smad4, Smad6, and Smad7) in the intestinal mucosa of 72 patients with UC treated with Kuijie Granule for 60 days. Colonic tissues were obtained by a virtual colonoscopy guided biopsy before and after Kuijie Granule treatment followed by pathological analysis and quantitative analysis of TGF-*β*/Smads using immunohistochemistry. Kuijie Granule treatment significantly improved symptoms associated with UC, which include diarrhea, mucus production, pus and blood in stool, abdominal pain and distention, and tenesmus. The clinical benefit of Kuijie Granule treatment correlated with decreased expression of TGF-*β*1 and Smad7 and increased expression of TGF-*β*RII and Smad4. These clinical results indicate that Kuijie Granule can alleviate the symptoms associated with UC and modulate TGF-*β*/Smads signaling.

## 1. Introduction

Ulcerative colitis (UC) is considered an autoimmune disease influenced by several factors including genetics and environmental factors. In western countries, the annual incidence of UC is 79.268/100,000 [1] and in China approximately 11.62/100,000 [2]; however, in recent years, the incidence of UC has steadily increased [3]. Individuals with UC commonly begin to experience symptoms during early adulthood with a similar incidence between males and females [4]. UC is a chronic or long lasting disease [5] that causes many sporadic symptoms, including abdominal pain, diarrhea, and bloody mucopurulent stool [6]. The treatment goal of UC is to induce remission and prevent recurrence [7].

While the underlying etiology of UC has not yet been elucidated, the most widely held hypothesis on the pathogenesis of UC is immune hypersensitivity. Activation of cells belonging to both the innate and acquired immune system results in secretion of cytokines, such as IL-1, IL-6, IL-8, TNF-*β*, and IFN-*γ* that mediate the pathogenesis of UC. Furthermore, there are cytokines that have anti-inflammatory effects and maintain homeostasis in the colon, such as IL-4, IL-10, and TGF-*β* that are diminished in patients with UC. It is this imbalance between proinflammatory cytokines and anti-inflammatory cytokines that is associated with the symptoms associated with UC [8]. Altered TGF-*β*/Smads signaling pathway has been implicated contributing to the cytokine imbalance associated with UC and its progression [9]. Kuijie Granule, a prescription widely prescribed in China, has antibiosis, anti-inflammatory, and antidiarrheal effects and is used in the treatment of UC.

To investigate whether the clinical benefit seen with Kuijie Granule treatment is associated with the TGF-*β*/Smads signaling pathway, we assessed the expression of several related proteins in colonic samples collected from patients with UC that were treated with Kuijie Granule. We found that treatment with Kuijie Granule resulted in decreased expression of TGF-*β*1 and Smad7 and an increased expression of TGF-*β*RII and Smad4 with no significant change in Smad2 and Smad6 expression. These results suggest a potential mechanism underlying the effectiveness of Kuijie Granule in alleviating the symptoms associated with UC.

## 2. Materials and Methods

### 2.1. Sample Collection

Samples were collected from August 2012 to August 2014 at Taishan Medical University Affiliated Hospital located in Taishan, Tai'an, Shandong, China. According to practice guidelines for the 2010 World gastroenterology organization on the diagnosis and treatment of inflammatory bowel disease [10], diagnostic criteria are as follows: (1) clinical manifestations: tenesmus, often a small amount of diarrhea, and hematochezia; (2) endoscopy: diffuse colon, superficial inflammatory, involving the rectum, flaky, superficial erosions, and ulcers, and spontaneous hemorrhage; (3) tissue pathology: mucosa or submucosa diffuse inflammatory and crypt structure deformation. A total of 72 patients were identified for inclusion. Patients undergoing Kuijie Granule treatment underwent a virtual guided biopsy before and after treatment. Samples were fixed immediately in 10% formaldehyde and then embedded in wax block to avoid hemorrhagic and necrotic tissue. Block was sectioned at 5 *μ*m thickness on glass slides for pathological and immunohistochemical analysis. Only patients with pathological diagnosis were included for analysis. Exclusion criteria included malignancy, other specific inflammatory disease, and patients currently on treatment regimen that includes hormonal, antibiotics, or other medication.

### 2.2. Kuijie Granule

Kuijie Granule is a herbal prescription Chinese medicine that includes dark plum (15 g), magnolia vine (10 g), rhizoma coptidis (15 g), herba portulacae (30 g), fructus* Rosae laevigatae* (20 g),* Agastache rugosus* (15 g), gallnut (15 g), semen nelumbinis (30 g), semen euryales (20 g), fructus psoraleae (20 g), roasted radix* Puerariae* (30 g), and liquorice (10 g). All ingredients are made into traditional Chinese medicine granule and the weight of granule is 30.25 g. 30.25 g is loaded with an average of two packages and patient takes a single package (15.125 g) each time, PO (per os), twice daily. One course of treatment is considered to be 10 days. Patients in current study received a total of six courses.

### 2.3. Immunohistochemistry

#### 2.3.1. Reagents

Rabbit anti-TGF-*β*1, rabbit anti-TGF-*β*RII, rabbit anti-Smad2, rabbit anti-Smad4, rabbit anti-Smad6, and rabbit anti-Smad7 monoclonal antibody were purchased from Wuhan Boster Biological Technology, Ltd. (Wuhan, China). Ready-to-use immunohistochemical ElivisionTM kit plus polymerase HRP conjugate and amplifying reagents were purchased from Fuzhou Maixin Biotechnology Development Company, Ltd. (Fujian, China).

#### 2.3.2. Staining

Sections were deparaffinized and rehydrated and placed in a low-pH citrate buffer and subjected to antigen retrieval via pressure chamber for 30 min and allowed to cool to room temperature. Sections were incubated overnight at 4°C in primary antibody diluted in 1 : 50. Slides were incubated in amplifying reagent for 10–15 min at room temperature followed by incubation of polymerase conjugates for 15 min. The resulting activity was detected with diaminobenzidine (DAB) and counterstained in hematoxylin.

#### 2.3.3. Positive and Negative Controls

The positive controls used for immunohistochemistry are as follows. Colon cancer tissue was used as the positive control for TGF-*β*1 and Smad6, breast cancer tissue for TGF-*β*RII, normal gastric tissue for Smad2, and normal colorectal tissue for Smad4 and Smad7. PBS was used as a negative control for all markers used in study.

#### 2.3.4. Evaluation

Immunohistochemistry was evaluated using light microscopy using stereological methods described in Yoshikawa et al. [11]. Briefly, yellow or brownish yellow granular cells in the nucleus and/or cytoplasm were regarded as positive. To calculate the percentage of positive stained cells in a given sample, 5 random 10 × 10 micron fields were evaluated and scored based on cells positive for that specific marker. A score was given based on the percentage of positive cells, 1 point: (−), 0~10%; 2 points: (+), 11%~25%; 3 points: (++), 26%~75%; and 4 points: (+++), 76%~100%.

### 2.4. Evaluation of Clinical Improvement

At the conclusion of the 60-day treatment with Kuijie Granule, patient symptoms were evaluated according to criteria established by the guiding principle of clinical research on new drugs of traditional Chinese medicine [12]. The evaluation method is as follows: ask about patients' clinical symptoms including diarrhea, purulent blood, mucus, abdominal pain, abdominal distention, and tenesmus and then contrast the scores of clinical symptoms. Scoring criteria are shown as follows: 0 means no clinical symptoms; 3 means minor symptoms with small effects on QoL (Quality of life), which was assessed by inflammatory bowel disease questionnaire [13]; 6 means moderate clinical symptoms with significant impairment in daily functioning; 9 means severe clinical symptoms with patients severely debilitated in daily functioning. Symptoms are grouped into 3 general categories: (1) resolved, clinical symptoms are no longer experienced with inconspicuous pathology in the intestinal mucosa revealed by colonoscopy; (2) improved, clinical symptoms alleviated with some underlying and intestinal mucosa lesions present by colonoscopy with significant improvement; (3) invalid, no improvement in both clinical symptoms or by colonoscopy.

### 2.5. Statistical Analysis

Using SPSS 18.0 statistical software for data analysis, enumeration data use rank-sum test and measurement data is expressed in mean differences ± standard deviation (*x* ± *s*) and by using *t*-test. *P* < 0.05 was considered to be statistically significant.

## 3. Results

### 3.1. Baseline Characteristics

Samples were collected from individuals aged between 19 and 65 with the median age of 34 years. Men accounted for 40/72 (55.6%) of cases with women accounting for 32/72 (44.4%) of cases. In terms of the lesion location in the colon, 28 cases were in the rectum, 30 cases were in the sigmoid, and the remaining 14 cases were located either in ascending, transverse, or descending colon. Pathological analysis suggested an active period in 54 cases and an inactive period in 18 cases. In 56/72 cases, patients were experiencing multiple symptoms at the start of the study, such as abdominal pain, diarrhea, and mucus/purulent blood. In 16/72 cases, patients' symptoms were limited to abdominal pain.

### 3.2. Clinical Effectiveness of Kuijie Granule Treatment

**Figure 1:**
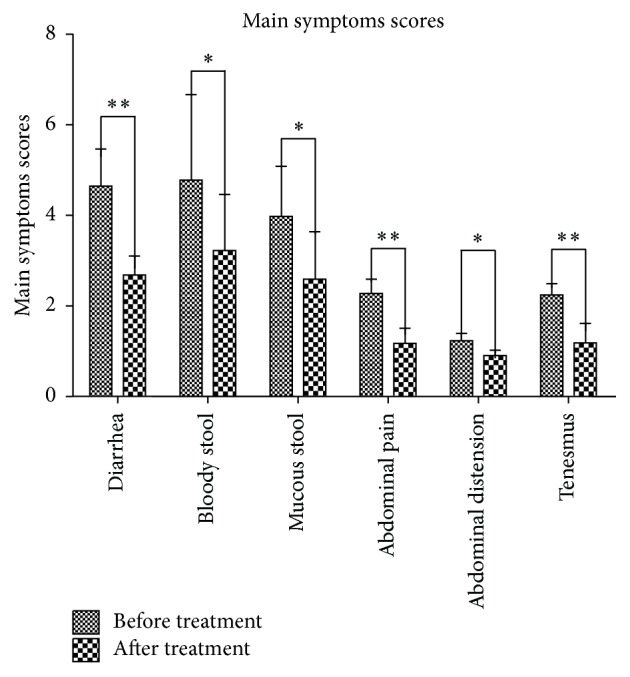
Kuijie Granule decreases the clinical symptoms of UC. Clinical symptoms associated with UC, diarrhea, mucous bloody stool, abdominal pain, abdominal distention, and tenesmus were evaluated in 72 UC patients before and after Kuijie Granule treatment for 6 courses. Symptoms were scored by the following specific criteria: 0, no clinical symptoms; 3, minor symptoms with small effects on QOL; 6, moderate clinical symptoms with significant impairment in daily functioning; 9, severe clinical symptoms; patients are severely debilitated in terms of daily functioning. ^*∗*^ *P* < 0.05, ^*∗∗*^ *P* < 0.01 indicate a significant difference before and after Kuijie Granule treatment. QOL = Quality of Life.

Clinical evaluation for the 72 patients treated with Kuijie was conducted as described in Methods. Symptoms evaluated were diarrhea, mucous bloody stool, abdominal pain, abdominal distention, and tenesmus. The symptoms associated with UC were resolved in 13 cases (18.1%), improved in 43 cases (59.7%), and invalid in 16 cases (22.2%) with a total effective rate of 77.8%. There have been significant differences before and after Kuijie Granule treatment (*P* < 0.05 or *P* < 0.01) (as shown in Figure 1).

### 3.3. Immunohistochemical Evaluation of TGF-*β*/Smads Signaling Pathway in Colon Samples following Kuijie Granule Treatment

#### 3.3.1. Transforming Growth Factor Beta 1 (TGF-*β*1)

**Figure 2:**
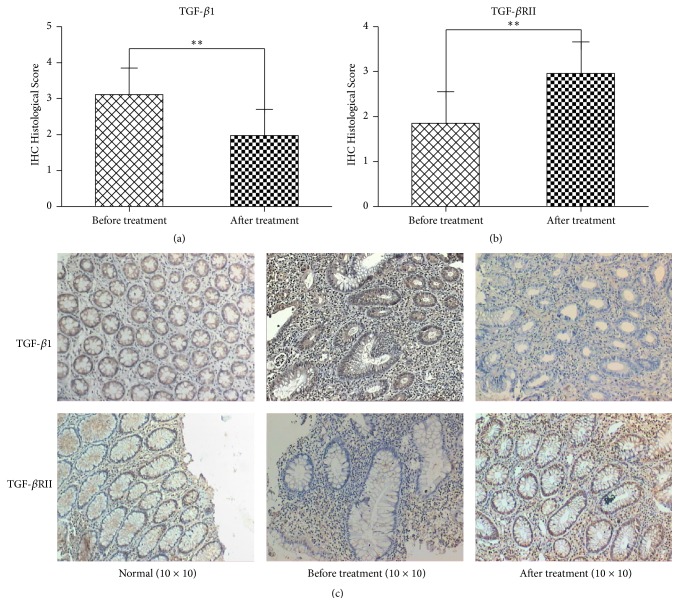
Kuijie Granule decreases the expression of transforming growth factor beta 1 (TGF-*β*1) and increases beta-receptor II (TGF-*β*RII) in colon tissue of patients with UC. (a) and (b) show the data analysis results for TGF-*β*1 and TGF-*β*RII expression; there exist significant differences before and after Kuijie Granule treatment (^*∗∗*^ *P* < 0.01); *n* = 72. ICH Histological Score means the integration of patients in TGF-*β*1 and TGF-*β*RII expression which come from the percentage of positive cells, 1 point: 0~10%, 2 points: 11%~25%, 3 points: 26%~75%, and 4 points: 76%~100%. (c) Representative images of the microscopic images obtained from colon tissues of patients with UC staining with anti-TGF-*β*1 and anti-TGF-*β*RII.

TGFs are key regulatory peptides in the intestine that modulate mucosal cell populations that are critical to UC. In normal intestinal mucosa, TGF-*β*1 is weakly expressed predominately in the cytoplasm. The expression of TGF-*β*1 is closely related to the degree of inflammation in UC. The expression of TGF-*β*1 during the active stage is significantly higher than that during the remission stage [14]. We were able to demonstrate that patients with active UC had an increased expression of TGF-*β*1 compared to patients in remission. The expression of TGF-*β*1 was (−) 1/72, (+) 10/72, (++) 39/72, and (+++) 22/72, respectively, before treatment. Following treatment with Kuijie Granule, we found a significant decrease in the expression of TGF-*β*1 (*t* = 21.06, *P* < 0.01) (Figures 2(a) and 2(c)), which was (−) 17/72, (+) 41/72, (++) 12/72, and (+++) 2/72, respectively. The expression was diffuse predominately in the cytoplasm with some nuclear staining in large cells.

#### 3.3.2. Transforming Growth Factor, Beta-Receptor II (TGF-*β*RII)

TGF-*β* binds to the TGF-*β*RII triggering the kinase activity of the cytoplasmic domain that in turn activates TGF-*β*RI. The complex results in nuclear translocation of Smad molecules and gene transcription amplifying the TGF-*β* signal [15]. It is believed that expression of TGF-*β*RII is an indication of immune system tolerance thus limiting the inflammatory reaction during inflammatory bowel disease [16]. TGF-*β*RII is a transmembrane protein that is expressed in cytoplasm with some nuclear expression. The expression of TGF-*β*RII was (−) 23/72, (+) 38/72, (++) 10/72, and (+++) 1/72, respectively. Kuijie Granule treatment increased the expression of TGF-*β*RII in the intestinal mucosa (*t* = −21.94, *P* < 0.01), which was increased, respectively, as follows: (−) 2/72, (+) 13/72, (++) 43/72, and (+++) 14/72 (as shown in Figures 2(b) and 2(c)).

#### 3.3.3. Smad Proteins

**Figure 3:**
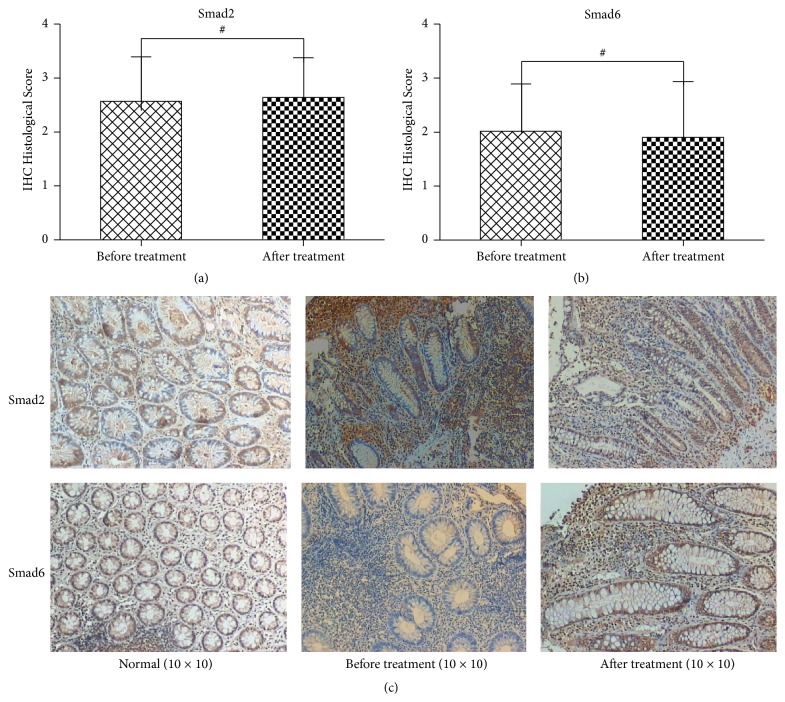
Kuijie Granule does not affect the expression of Smad2 and Smad6 in colon tissue of patients with UC. (a) and (b) show the data analysis results for Smad2 and Smad6 expression; they have no significant differences before and after Kuijie Granule treatment (^#^ *P* > 0.05); *n* = 72. ICH Histological Score means the integration of patients in Smad2 and Smad6 expression which come from the percentage of positive cells, 1 point: 0~10%, 2 points: 11%~25%, 3 points: 26%~75%, and 4 points: 76%~100%. (c) Representative images of the microscopic images obtained from colon tissues of patients with UC staining with anti-Smad2 and anti-Smad6.

The Smad proteins are the intracellular effectors that mediate the TGF-*β* signaling cascade. Smad proteins are activated by the TGF-*β* receptor and translocate into the nucleus where they regulate transcription; however, the combinational interaction of the heterodimer and Smad complexes determines the nature of the response. For example, the combination of Smad2 and Smad4 suppresses the secretion of proinflammatory factors [17]. We found that the expression of Smad2 was (−) 7/72, (+) 25/72, (++) 32/72, and (+++) 8/72, respectively, while, after Kuijie Granule treatment, its expression was (−) 5/72, (+) 22/72, (++) 39/72, and (+++) 6/72, respectively. There were no significant changes in the expression of Smad2 with Kuijie Granule treatment (*t* = −1.69, *P* > 0.05) (Figures 3(a) and 3(c)). The expression of Smad6 was (−) 23/72, (+) 29/72, (++) 16/72, and (+++) 4/72, respectively, while, after Kuijie Granule treatment, its expressions were still with no big changes (*t* = 1.92, *P* > 0.05), which were (−) 33/72, (+) 22/72, (++) 8/72, and (+++) 9/72 (as shown in Figures 3(b) and 3(c)). Smad6 can inhibit the phosphorylation of Smad2 effectively blocking the signal transduction and suppressing the inflammatory reaction [18].

**Figure 4:**
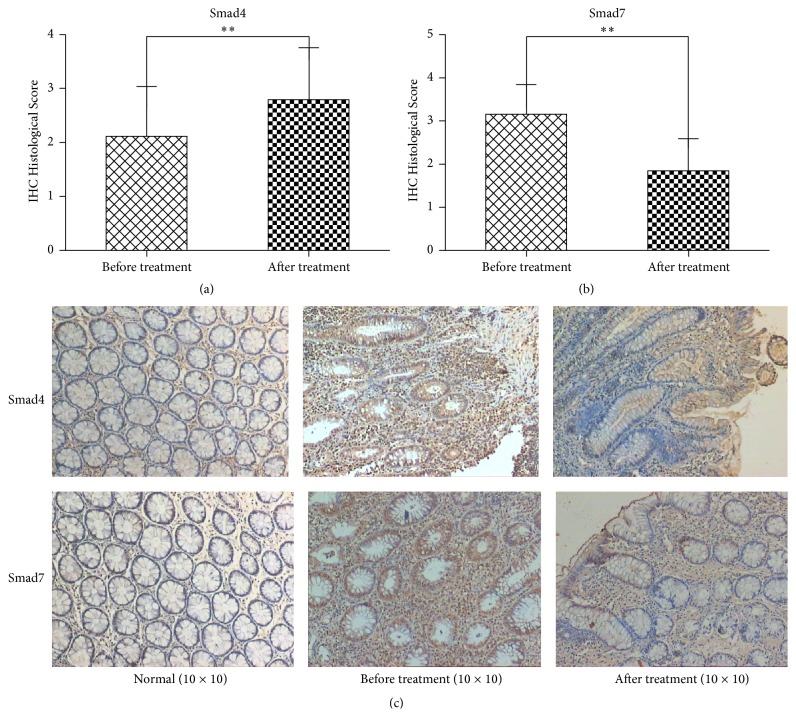
Kuijie Granule increases the expression of Smad4 and decreases Smad7 in colon tissue of patients with UC. (a) and (b) show the data analysis results for Smad4 and Smad7 expression; there exist significant differences before and after Kuijie Granule treatment (^*∗∗*^ *P* < 0.01); *n* = 72. ICH Histological Score means the integration of patients in Smad4 and Smad7 expression which come from the percentage of positive cells, 1 point: 0~10%, 2 points: 11%~25%, 3 points: 26%~75%, and 4 points: 76%~100%. (c) Representative images of the microscopic images obtained from colon tissues of patients with UC staining with anti-Smad4 and anti-Smad7.

We also found that the expressions of Smad4 were (−) 21/72, (+) 28/72, (++) 17/72, and (+++) 6/72, respectively, while, after Kuijie Granule treatment, its expressions were significantly increased as follows: 9/72, (+) 15/72, (++) 30/72, and (+++) 18/72 (*t* = −12.30, *P* < 0.01) (Figures 4(a) and 4(c)). Smad7 is also a major negative regulatory protein in the TGF-*β*1 signaling pathway, which blocks signal transduction by regulating Smad phosphorylation activation and reduces the inflammatory reaction [19]. We found that the expressions of Smad7 were (−) 1/72, (+) 9/72, (++) 40/72, and (+++) 22/72, respectively, while, after Kuijie Granule treatment, its expressions were significantly increased as follows: (−) 25/72, (+) 34/72, (++) 12/72, and (+++) 1/72 (*t* = 21.26, *P* < 0.01) (Figures 4(b) and 4(c)).

## 4. Discussion

In recent years, the incidence of ulcerative colitis (UC) has been steadily on the rise. The TGF-*β*/Smads signal transduction pathway (TGF-*β*1, TGF-*β*RII, Smad2, Smad4, Smad6, and Smad7) has been implicated in the pathogenesis of UC [20]. TGF-*β*/Smads signal transduction pathways consisted of TGF-*β* super family, TGF-*β* receptors, Smads protein family, and its nuclear transcription regulator of signaling pathways that regulate cell growth [21]. TGF-*β* is a pleiotropic cytokine with potent regulatory and inflammatory activity that can promote angiogenesis, inhibit immune responses, and even promote the growth, invasion, and metastasis of tumors [22]. TGF-*β* is ubiquitously expressed in intestinal epithelial cells, fibroblasts cells, and T-cells [23] and is tightly regulated [24]. Xu, et al. [25] demonstrated utilizing an ulcerative colitis rat model that TGF-*β*1 was significantly higher during active UC and correlated with disease severity. They expound that TGF-*β*1 plays an important role in the process of the ulcerative colitis development, and the expression of TGF-*β*1 is closely related to the severity of UC. TGF-*β*RII may perform self-phosphorylation and then activates TGF-*β*RI which can amplify TGF-*β* signal [15]. Thus, the combination of TGF-*β*1 and TGF-*β*RII is the further development of illness. In the current study, the expression level of TGF-*β*1 is lower after Kuijie Granule treatment compared to that before the treatment (*P* < 0.01), and the expression level of TGF-*β*RII is higher after Kuijie Granule treatment than that before the treatment (*P* < 0.01). These results suggest that Kuijie Granule can block the combination of TGF-*β*1 and TGF-*β*RII in a certain degree and then reduce the risk of further development and carcinogenesis of UC.

TGF-*β*1 binding to type II receptor results in activation of the Smad family proteins, one of the main effector molecules of TGF-*β* that regulates gene transcription [26, 27]. There exist at least 9 members of the Smads protein family, which is divided into 3 categories according to their functions [28, 29]: (1) pathway-restricted Smads (R-Smads), which includes Smad1, Smad2, Smad3, Smad5, Smad8, and Smad9; (2) common-mediator Smad (Co-Smad), including Smad4; and (3) inhibitor Smads (I-Smads), including Smad6 and Smad7. When TGF-*β* combines with its receptor of T*β*RII and T*β*RI, it makes T*β*RI phosphorylation and can be combined with R-Smads in the cytoplasm briefly. MH2 of the R-Smads-COOH contains a characteristic Ser-Ser-X-Ser sequence (SSXS motif), its Ser residues can be phosphorylated by T*β*RI, thereby activating Co-Smad. Phosphorylated Smad2 or Smad3 in combination with Smad4 forms heterogeneous complex, which can enter the nucleus and regulate transcriptional activity [30]. Smad6 and Smad7 have similar structure to R-smads; they can compete with Smad2 and Smad3 to combine with T*β*R or Smad4, inhibit the phosphorylation of Smad2 and Smad3, and block the signals of the TGF-*β* transferring into the nucleus and then block the transmitting of the signals [31–35].

In the current study, Smad2 and Smad6 expressions have no obvious changes before and after Kuijie Granule treatment (*P* > 0.05), which indicates that Kuijie Granule has no effect on Smad2 and Smad6. The positive expression of Smad4 is higher than that before Kuijie Granule treatment (*P* < 0.01), which shows that Kuijie Granule helps in increasing the positive expression of Smad4 which is very important for inhibiting the development of UC to tumor. The positive expression of Smad7 is lower than that before Kuijie Granule treatment (*P* < 0.01), which shows that the lower expression of Smad7 has a positive effect on blocking signal transduction of the TGF-*β* and preventing further progression of UC.

Traditional Chinese medicine holds that the main pathogenesis of UC is damp-heat and blood stasis which block blood vessel of bowel, and then intestinal vascular ischemia induces local necrosis, hemorrhage, and appearing purulent bloody stool. With the extension of course of disease, genuine qi was damaged and the pathogenesis of UC cannot be removed, which makes it difficult to treat UC. So according to the TCM concepts, the principle for the treatment of UC should be clearing away heat and eliminating dampness, warming Yang, convergence, and antidiarrheal. Kuijie Granule is composed of Chinese herbals based on the above theory. In this experiment, 72 patients with UC were treated with Kuijie Granule for 60 days, and the symptoms for UC patients, such as diarrhea, mucus stool, purulent bloody stool, abdominal distension, abdominal pain, and tenesmus, were significantly improved (*P* < 0.05, *P* < 0.01); the total effective rate was 77.8%; these results indicate that Kuijie Granule has a good clinical effect on UC. Kuijie Granule can decrease the positive expression of TGF-*β*1, TGF-*β*RII, and Smad7 and increase the positive expression of Smad4. We speculate that Kuijie Granule can improve the clinical symptoms of UC by adjusting the TGF-*β*/Smads signal transduction pathway.

**Figure 5:**
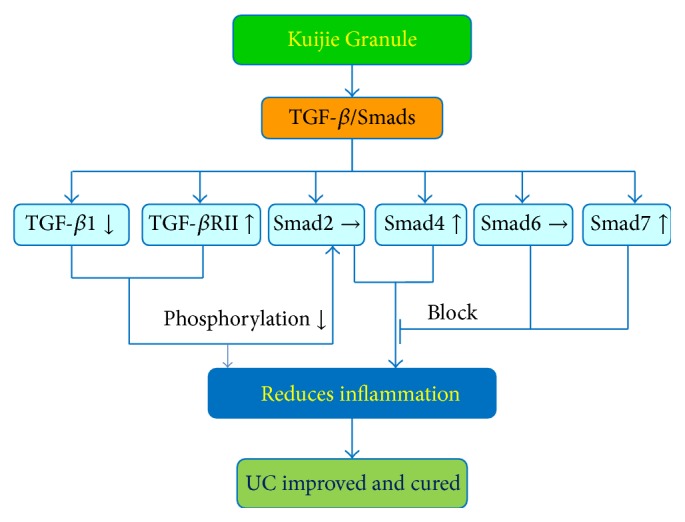
Mechanisms of Kuijie Granule in suppressing ulcerative colitis. Kuijie Granule can improve the clinical symptoms (diarrhea, mucous bloody stool, abdominal pain, abdominal distention, and tenesmus) in UC patients by regulating TGF-*β*/Smads signal transduction pathway. Kuijie Granule decreased the expression of TGF-*β*1, increased the expression of TGF-*β*RII, Smad4, and Smad7, and has no effect on Smad2 and Smad6 expression; then, the decreasing of the phosphorylation of anti-inflammation signal proteins and the blocking of the expression of inflammation signal proteins finally reduced inflammation reaction to improve or cure UC. Interactions leading to activation of molecular targets are indicated by arrows; those that are inhibited are indicated by a bar.

**Figure 6:**
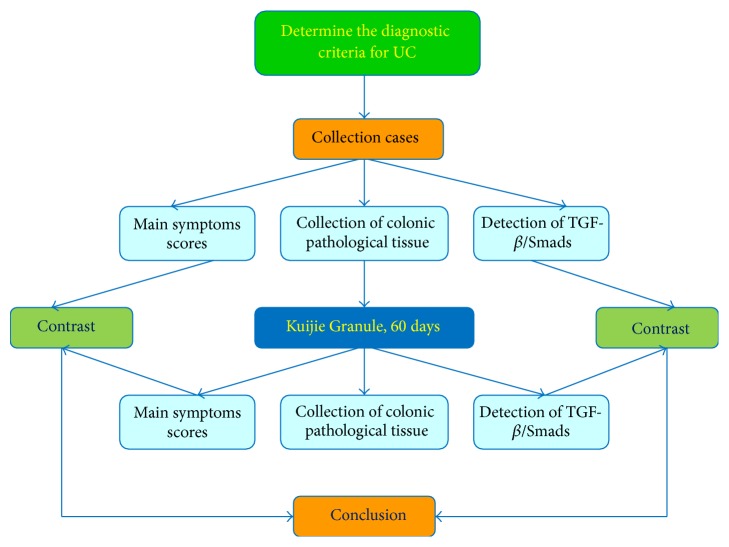
Study flow chart of Kuijie Granule for ulcerative colitis. In this study, we take steps as follows: (1) determine the diagnostic criteria for UC; (2) collect the cases and score the main symptoms; (3) collect the colonic pathological tissue to detect and analyze the expression of TGF-*β*/Smads, which include TGF-*β*1, TGF-*β*RII, Smad2, Smad4, Smad6, and Smad7; (4) treat UC patients with Kuijie Granule for 60 days; (5) repeat steps (2) and (3); (6) compare the main symptoms scores and TGF-*β*/Smads expression before and after Kuijie Granule treatment; (7) get the conclusions.

In conclusion, Kuijie Granule is a herbal prescription medication that is effective in treating the symptoms associated with UC with a clinical improvement of 77.8%, which correlated with changes in the expression of TGF-*β*1, TGF-*β*RII, Smad4, and Smad7 in the TGF-*β*/Smads signal transduction pathway (as shown in Figures 5 and 6). Future studies with larger sample size and further mechanistic studies will be necessary to explain the direct relationship between Kuijie Granule and TGF-*β*/Smads signal transduction pathway. Kuijie Granule is a traditional prescription which we have used in clinical practice for UC treatment, in which effective component and active ingredients are complex. We speculate that the effective components and active ingredients of Kuijie Granule may include organic acids (such as citric acid, malic acid), alkaloids (such as berberine), and tannic acids (such as Chinese gallotannin). It is very necessary to get the active ingredients clearly in the following studies.
